# Lehrbuch Der Krankheiten Der Weiblichen Sexualorgane

**Published:** 1857-11

**Authors:** 


ART. II.—*Lehrbuch der Krankheiten der Weiblichen Sexualorgane,* von Dr. F. W. SCANZONI, K. Bayer, Hofrath, Professor der Medecin an der K. Universität zu Würzburg. Wien, 1857; pp. 570.

*Text-book of Diseases of the Female Sexual Organs,* by Dr. F. W. SCANZONI, Prof. of Medicine in the Royal University at Wurzburg. Vienna, 1857.

WITHIN the last three decennial periods progress has been made in the anatomy, physiology, and pathology of the female generative organs, which challenges comparison with that made in any other department of medical inquiry. If it is a fact, as stated by one of the latest and best writers upon the subject,* that "thirty years ago the structure, functions, and diseases of the female generative organs were almost unknown," we have cause for congratulation on the increased knowledge we possess of these subjects, and a debt of gratitude to render to those who have, with so much perseverance, industry, and talent, investigated these interesting and important subjects.

Although an enumeration of those who have borne the chief part in this marked improvement would include some of those whose names are highest in the list of professional honor, and many who are renowned for their careful observation, profound research, and sound reasoning, there are many subjects in the department of our science yet unsettled—many upon which there exists a wide difference of opinion, and perhaps a wider difference than is usual in medical questions. This fact is not surprising when we consider the peculiar difficulties naturally attending the observation of the living patient, both as to the diseased conditions and the changes produced in them by remedies, and the few who are placed in situations where they can examine after death those changes produced by disease and by remedies during life.

Amid the conflicting opinions presented in books in our own language upon the importance of inflammation of a certain portion of the sexual organs of woman in deranging her general health, upon the use of certain mechanical contrivances for the cure of certain derangements of position of the organs, upon the frequency of the occurrence of ulceration, the value of caustics as a means of cure, and of the speculum as a means of diagnosis—amid the differences of opinion, we say, which prevail upon these subjects, it is well to look abroad for additional testimony on the questions at issue. Our science is bounded by no territorial lines; its teachings are confined to no exclusive dialect; and one may derive both

* West on the Os Uteri.

pleasure and profit from perusing the works of our professional brethren in other languages, without claiming that they are better or more reliable than those of our own.

We look upon the work before us as one worthy of being examined upon the debated points. The author is a man of note in a country which has produced as many eminent obstetricians and gynakologists as any other in the world—the country of Nægelé, Busch, the Siebolds, Carus, Osiander, Bischoff, Wigand, Kilian, and Kiwisch. His large and complete treatise on obstetrics has rapidly passed through three editions; and this means a great deal more than the same fact in regard to a work in this country. At present he is connected with a university of excellent standing, and one which has the honor of having attached to it another name well known to the medical profession throughout the world,—that of Kölliker, Professor of Microscopical Anatomy.

In the preface, the author lays before us the aim and objects of the work, and the reasons which influenced him in undertaking it. Of the latter, the principal one is stated to be, that the literature of Germany, while it contains a large number of voluminous treatises upon the subject, yet lacks a compendious work adapted at once to the wants of the student and the practitioner. This want he seeks to supply; for in the size and number of the volumes devoted to this subject, he sees an explanation of the fact, that a thorough knowledge of diseases of women among the practical physicians of his country "is one of the greatest rarities." He expresses a confidence in his fitness for the task he has undertaken, by ten years' devotion to the subject, during which time he was in the Lying-in Hospital at Prague, and at the head of the female wards of the general hospital of the same city; he regrets the loss of these rich fields of observation by his removal to Wurzburg, yet claims an extensive private practice there besides the hospital advantages. In the execution of the work, he assures his readers that they shall have the result of his own observations, and no mere compilations; he, therefore, apologizes for what may otherwise appear the neglect of the observations of other writers, and seeks to supply in part the omission by a general and special bibliography. We cannot but regret this omission upon many most interesting subjects; yet by it the views of the author are more clearly presented, and the value of the work as an original one is increased. Following the preface are three or four pages of general bibliography, containing the titles of the principal works upon the subject from the time of Hippocrates.

The work is divided into seven sections, according to the anatomical division of the sexual organs. We proceed to examine such points in each division as are of importance, and to present those views of the author which we may judge likely to be of value or interest to readers in this country.

The opening chapters of the work are occupied with general observations on the symptoms produced by diseases of the uterus, both local and general. The facts are briefly stated which may suffice for the practitioner, but they are not explained sufficiently for the student to profit by them. The second chapter introduces us to the examination of the diseased uterus. In accomplishing this, he thinks no instrument should supersede the sense of touch, and that he who is not expert at recognizing diseased conditions by this means, will do but little better with the speculum. While his estimation of this instrument, as a means of diagnosis, is far from being as high as many writers in our own language, he yet considers it to be indispensable in many cases. From his experience, and from the pains he takes to urge its value, yet without inculcating its use until after the usual means of diagnosis or of cure have failed, we should judge that it is but little resorted to by the profession in general in Germany. He gives it as his experience that no patient, not even the most modest, will refuse to allow its application, if its necessity and value are presented to her in a proper manner; and that where it is neglected, the fault "lies always in the good will of the physician." We would simply remark, that a difference of country may cause a difference of experience; and although keeping in mind the class found in the wards of a hospital, those who have frequented a German obstetrical *clinique* will bear us out in saying that there is a wide difference in the conduct of the sex of the two countries in the same social position. As to the kind of instrument preferred, the author expresses the opinion that no one will answer for all cases; the glass tube with metallic coating is preferred, as being most generally useful; but as it is not manufactured in Germany, he recommends some of the many-bladed metallic instruments, although "the cases in which they are more valuable than the other are very rare indeed."

The author does not belong to the class of those who deny altogether the utility of the uterine sound as a means of diagnosis; and he is as widely removed from those who advocate its general and almost indiscriminate employment. We will allow him to speak for himself:

"We have no intention of absolutely denying the usefulness of the sound for certain diagnostic and therapeutic objects; yet an extensive experience with the instrument during a period of many years, has forced the conviction upon us, that the advantages which were promised from its use in the beginning are not in truth nearly so great and numerous as were assumed for it; and although there are yet some gynakologists who maintain that an examination with the sound is an indispensable condition of a complete diagnosis of most diseases of the uterus, yet we are convinced that this view will, within a short time, give place to a more just one.

"The experience that the use of the uterine sound is by no means so devoid of danger as some would have us believe, will contribute in the highest degree to this result; for however much practice and dexterity one may have had in the use of this instrument, yet there will nevertheless be cases where its introduction into the cavity of the uterus is accompanied with considerable difficulty, and more or less serious injuries of the uterine mucous membrane may be occasioned by it. Since it is known that abortion has been repeatedly occasioned by the use of the sound, even in the hands of the most practised and renowned gynakologists—that the most violent spasmodic pains [die heftigsten uterinalkoliken], profuse hemorrhages, and even dangerous uterine and peritoneal inflammations have followed its application—so should we be very cautious about resorting to it, even if it could render us the most important assistance in diagnosis,—much more so when, as we firmly believe, it is of only subordinate value; for there is not the slightest doubt that the diagnosis of all diseased conditions of the genital organs, which cannot be made by other modes of examination, cannot also be made, except in the rarest cases, by means of the uterine sound."

The author is not alone in these opinions upon the use of this instrument. Among others, we may mention a reviewer of "Simpson's Obstetric Memoirs and Contributions," who has seen extreme pain and suffering, occasioned by a persistent hemorrhage, follow its application, and believes that great care and judgment are required in its use.* West, in his "Lectures on Diseases of Females," just published, bears testimony to the fact that the introduction of the sound occasions pain in the "majority of instances;" yet his opinion of its value and harmlessness is much higher than Scanzoni's.

The author bears testimony to the high value of Simpson's sponge-tents for dilating the cervix uteri, as an aid in diagnosis, and directs their application in many places through the work.

In the chapter upon general therapeutics, regret is expressed at the extent to which general treatment has been neglected, in favor of local, in female complaints; yet the author does not think the former can ever entirely supersede the latter. In the use of depletion, he thinks nothing can be a substitute for the direct withdrawal of blood from the organ affected, and prefers the application of leeches to the uterus to scarification. The different caustics and different forms of their application are mentioned, and he recommends the actual cautery for "carcinomatous ulcers, and those which have obstinately resisted the application of other means." He bears testimony to the small amount of pain it causes, but, we are sorry to say, recommends its secret employment. He makes some excuse to the

* British and Foreign Medico-Chirurgical Review, October, 1855, p. 331.

patient, and only informs her of the fact after she has recovered from the effects of the chloroform. He introduces a new article in this class to the attention of the profession—one which we do not think will be very soon or very often used in this country, being nothing more nor less than *melted sealing-wax!*

"We cannot here omit calling attention to a mode of treatment which we have lately often applied, and indeed with the best effects, to obstinate ulcerations of the os uteri. This is the cauterization of the surface of the ulcer with melted sealing-wax. After the vaginal portion of the uterus is isolated by means of a horn speculum, a stick of sealing-wax is brought to the melting point, and its end immediately applied to the desired surface. This operation inspires far less horror than the actual cautery, and occasions a much more intensive and deeper effect than the application of all the various caustics of the pharmacopœia."

The application of caustics to the mucous membrane lining the cervix and cavity of the uterus is spoken of as matters of course. They are used either solid or in solution. For the application of the solid article, an instrument of Kiwisch's is preferred to the *porte-caustique* of Lallemand, from which, however, it differs but little. Injections are made first of warm water, then very weak solutions of the substance used, which are to be gradually increased in strength until the desired result is obtained. We confess we were surprised to find such proceedings recommended by an author who has urged the objections given above against the uterine sound. He does not deny the possibility of the injection penetrating the peritoneal cavity through the Fallopian tubes, but has never seen an instance where peritonitis has followed the proceeding; but he admits that intense pain and other unpleasant symptoms following it have often fallen under his observation.

"But we have not seen these unpleasant results follow since we have adopted the principle of never applying remedies in this form except when the width of the cervical canal and os uteri allowed to the injected fluid a rapid exit; farther, we always limit the quantity of the injection to half an ounce, or not exceeding an ounce, and in applying it carry the point of a properly shaped syringe to the fundus of the uterus, against which the stream breaks, and flows down the walls of the cavity and out at the os."

Medicated pessaries, consisting of fine sponge dipped in the required solution, are recommended, as also those consisting of the remedy incorporated with an ointment, of such a consistence that, when inclosed in a fine muslin bag, it can be carried up to the extremity of the vagina. Such a pessary would consist of about ʒij of a mixture of lard and wax, in the proportion of ʒvj of the former to ʒij or more of the latter, and the requisite amount of the medicine to be applied. In this manner opium and belladonna may be applied; also various astringents, iodine, and the iodides of potassium and lead.

The solution and absorption of these ointments are, however, not rapid enough to make them of service where the indication is to relieve severe pain. Here injections must take their place, or the end be attained by the local application of the vapor of chloroform. This is spoken of in high terms, and an instrument represented for its administration which is very simple, and its place may be easily supplied by any one. The author has not yet had sufficient experience to give a full opinion upon it, yet he has seen it render excellent service. Its effects are produced in about ten minutes when it succeeds; and in some cases where it has failed, "a rapid and favorable result" has followed the introduction of the vapor into the rectum.

The injection of various medicines into the vagina receives the consideration which its importance as a means of cure demands; and delineations are given of various instruments for the application of the uterine douche, a remedy which the author considers of the greatest value. His remarks upon the temperature of injections, and the effect of different degrees of force with which they may be introduced, are of so practical a nature that we cannot avoid introducing them, although the quotation is rather long.

"As a general rule, it will hold good that the flow of blood to the genital organs increases in proportion to the temperature of the injected fluid; this is shown by the increased warmth of the vagina, by the turgescence of its walls, by the increased secretion of its mucous membrane, and, after long continuance of the remedy, by a perceptible intumescence of those portions of the uterus within reach of the finger. The patient complains of a feeling of heaviness, heat, and fulness in the pelvis, which sometimes extends to the lumbar region; not seldom the influence of the warmth upon the general organism shows itself by an increased frequency of pulse, a sense of fulness in the head, palpitations of the heart, and sometimes even by a smart febrile movement. The exciting influence of the warm douche upon the sexual system is also shown in the breasts by the considerable swelling of these organs and darting pains in them, and by the swelling of the lymphatic vessels and glands of the axilla. But the most decided evidence of this increased congestion is given by the increased menstrual flow which is so often seen to follow the use of warm injections.

"The application of warmth is recognized as a remedy of decided value in aiding the absorption of exudations, and it is not therefore surprising that enlargements of the uterus, which have their origin in inflammatory exudations in its parenchyma, so frequently decrease under the application of the warm douche.

"The effects of the cold douche are limited, at least in so far as perceptible, to the parts with which the water comes in immediate contact. When an examination is made shortly after the use of the cold injection, the vagina is found narrowed from the stronger contraction of its walls, and the secretion of its mucous membrane generally diminished; if there had been some sinking of the uterus and the walls of the vagina, in the first half or whole hour after the use of the remedy, it is entirely abolished, or very much moderated, and the effect of the cold upon the uterus itself is evident from the fact that enlargements of it, depending upon chronic congestion of its parenchyma, decrease in a marked manner even after a short use of the douche.

"The force with which the injected fluid strikes the uterus essentially modifies the effect of the injection according to its greater or lesser degree, so that the congestion resulting from the use of the warm douche is greatly increased if the fluid is poured into the genitals with considerable force and in an unbroken stream, and even a cold injection applied in this manner will produce the same effect as a warm one applied with less force. The mechanical irritation here supplies the place of the high temperature, a fact which should be borne in mind in cases where cold injections are ordered for a hyperæmia of the genitals or to arrest a hemorrhage."

Under the head of "Pathology and Therapeutics of the Uterus," the first division opens with a chapter on the absence and malformations of the organ, and others follow on its rudimentary formations, the result of checked development, on its atrophy and hypertrophy, and other subjects, which pass over the whole ground in that complete and laborious manner characteristic of the German mind. The subject of flexions of the uterus occupies no less than forty pages of the work. His views upon this subject attracted a great deal of attention, but the importance of his conclusions have not yet been fully determined.

The bend in the uterus usually takes place at the junction of the cervix and body, the extent of deviation from the normal axis varying very much, sometimes being so great as to produce complete *atresia uterinæ*, of which he gives two cases, the patients both being old women. Virchow holds that flexions of the uterus are caused by adhesions which connect it to other organs, the result of peritoneal inflammations. From this view Scanzoni dissents, although he admits the frequency with which these connections are met, and his best reason, among many, for dissenting from so distinguished an authority is that he has observed these deviations of shape in the organ where "not the slightest trace of peritoneal adhesions was to be found." He states that in these cases the cervical portion is always found more or less flattened from before backward, and its parenchyma evidently changed, the latter being less resistant, more spongy and weak than natural. Judging from a single microscopical examination, which we think small enough for a writer who has enjoyed such opportunities, he thinks that fatty degeneration of the walls of the organ is the cause of its altered shape, yet is obliged to leave the question to the pathological anatomists for decision. In this view, concerning structural change taking place at the point of flexion, he is sustained by several other writers. Although he is aware that these variations from the usual shape of the uterus are frequently found before puberty and after the cessation of menstruation; yet he thinks the absence of its periodical congestion prevents any symptoms being developed, and he finds in those conditions produced in the organs by pregnancy and child-bearing the chief causes of the accident. Too early marriage he looks upon as having a great influence in producing this as well as other diseases of the uterus, as also does too frequently repeated child-bearing.

In abortion he finds a prominent cause of the same. Of 252 births by 43 patients suffering from flexion, 12 were premature, and 44 abortions,— so that about 22 per cent. of the cases were interrupted in their course. He explains this influence by the statement of the fact that after abortion the involution of the uterus goes on relatively much slower than after labor, and by the much earlier period at which women resume their household duties after the accident than after the natural process.

The manner of termination of labor deserves examination in connection with the existence of flexion. Thus, of the 252 births mentioned above, 196 were at the full term; of these, 34 were terminated artificially, 14 by the forceps, 16 by version, and in four cases unknown. In thirteen cases version was necessary from transverse positions of the child, an "anomaly certainly occasioned by a greater or less degree of flabbiness of the uterine walls during the course of pregnancy;" and in many of the cases the forceps were necessary from insufficient pains.

> "Since it is certain that a delay in the involution of the organ after delivery follows where its power of contraction during labor is feeble, it is certainly not maintaining too much to say that flexions of the uterus are far more frequently observed in those who have been delivered artificially than in those who have brought forth naturally."

Of the 43 patients mentioned, 16 had been delivered of twins, and accidents during or after delivery had occurred in unusual proportion,— hemorrhage 64 times, inflammatory affections 24 times, and very severe after-pains 8 times.

Deficient involution of the uterus being, according to the author, the cause of these diseased conditions, he looks upon all those causes which produce the one as likely to produce the other. Leaving bed too soon after delivery he considers a frequent cause, it having been remarked in a large proportion of the cases he has seen. The influence of nursing the child is shown by the fact that 54 women suffering from flexion of the uterus had given birth to 196 children at full term, of which only 57 children had been suckled.

"Nobody will pretend to deny that the irritation of the nerves of the breast from suckling the child, by the more energetic contractions of the uterus it produces, not only occasions its present diminution, but essentially assists its entire and permanent involution; but when this favorable impulse is lacking, it is plain that the larger, heavier, and likewise flabby organ, more readily gives way to any external impulse, sinks over backwards or forwards, and a flexion is thus easily established."

The symptoms accompanying ante- and retro-flexions of the uterus are pains, menorrhagia, leucorrhœa, and difficulty in voiding the contents of the bladder and rectum, various derangements of digestion, and chlorosis. Derangement of menstruation in regard to quantity and time come first, then pain, difficulty of urination, and other symptoms following in order. But these, in the author's opinion, are only *accompanying* symptoms.

"Authors have, however, gone much too far when they have represented all these symptoms as the *immediate* consequence of the inflexions of the uterus. We would by no means deny that this evil is an important one for the economy of the whole body, yet we cannot avoid expressing the conviction that lately, when inflexions of the uterus have become a fashionable subject, and a hobby-horse for gynakologists, if we may so express ourselves, that lately the importance of the same has been by far over-estimated, and an influence ascribed to them, upon the sexual system, as well as upon the general organism, which they can only mediately exercise.

"We ourselves formerly, in the beginning of our gynakological practice, ranked with those who, like Kiwisch, Mayer, Simpson, Valleix, and others, could not estimate high enough the injurious influence of inflexions of the uterus upon the general health of the patient; we confess openly that it was only with difficulty that we abandoned a doctrine which is yet looked upon by a considerable number of able colleagues as one of the most important advances in the domain of uterine pathology. But, finally, we have been compelled to abandon our earlier convictions through numerous observations directly opposing them, and we now candidly express our opinion, *that inflexions of the uterus only attain a great importance, only have serious derangements of health following them, when some other organic change is associated with them.*"

The proofs of this statement are given at length, but may be briefly stated to be founded on the discovery he made of the presence of these deviations in the examination of patients who, during life, were under his observation, and in their last illness were under his care, and who yet had no symptoms of uterine disease; and of cases in which he satisfied himself during life of the existence of inflexion, and which was unaccompanied by anything more serious than a slightly increased mucous discharge. He looks upon the inflexions as sometimes the cause and sometimes the consequence of changes in the texture of the uterine walls.

In treating of the diagnosis of this disease, he again notices the uterine sound, and its value in effecting this object. The frequency with which he has seen injurious consequences follow attempts to introduce it into a flexed uterus leads him to reject its use, except in cases where all other means of diagnosis have failed, and such cases he thinks occur but very rarely.

"But it may be suggested that this instrument essentially assists those practitioners in diagnosis, who, not being professed gynakologists, would willingly avail themselves of an instrument which, although not indispensable to the experienced, is with them scarcely to be avoided. To this we would reply, that it is in just such hands that the sound is an extremely dangerous instrument, and that when such an one cannot arrive at the true state of affairs in other ways, he is least of all likely to do so by means of the sound, for the simple reason, that he who cannot diagnose an inflexion from a manual examination, can never, or at least only very seldom, indeed, succeed in passing the sound beyond the point of flexion."

The cure of this evil he considers as one of the most unpromising tasks the physician can undertake; it defies medicinal and mechanical means, because its progress is so gradual that attention is not called to it until organic changes of such a degree and nature have supervened as to place it beyond the reach of remedies. He has seen three cases of anteflexion where conception took place and gestation went to the full term. In two of these cases a perfect and permanent cure was the result, in the other the flexion returned after delivery in increased degree; but as the result of his own treatment he has not seen a single permanent cure.

He does not at all agree with the doctrines which have become so popular of late, in regard to the application of mechanical means for its cure. He has had experience only with the intra-uterine pessaries of Kiwisch, Valleix, and Detschy, but expresses himself plainly upon their merits in general:

"After all that we have seen of these instruments, we cannot give our assent to their use. We hold their application to be dangerous, useless, and, in consequence of many circumstances attending individual cases, impracticable and unreliable."

He refers to the journals as containing numerous instances of the dangerous effects following the application of this instrument, and testimony of the same character can be found in writers in our own language, especially in the works of Bennett and Tilt. After relating a case where the stem pessary caused peritonitis so severe that for several days life was in imminent danger, he says:

"Violent and very painful contractions of the uterus, and profuse hemorrhages from the irritated mucous membrane, we have repeatedly observed, but these circumstances would not have frightened us from a farther use of the instrument if we had gained the conviction that it can really, as maintained on many sides, render essential and permanent service in the cure of the disease. Unhappily, this is not the case. When one has seen women submit themselves for months together to this highly painful mode of treatment with the greatest patience and perseverance; when one has seen them, during this long time, renounce almost all social enjoyments, in order to be able to wear an instrument from which they confidently expected the cure of all their sufferings; when one has seen all this, after all these sacrifices on the part of the patient, and found no case in which relief was permanent after removal of the instrument, so will one have good reason to doubt its adaptation to the disease and its power of cure."

The author remarks, that it may be retorted upon him that others have had such success with this mode of treatment that his failure must be attributed to his unskilfulness in the use of the instrument. To this he replies, that he has followed Kiwisch in the city where he now resides, Wurzburg, and that he has now in his wards twelve women who have worn the apparatus, and been under the personal care of that celebrated man, and that they are still suffering under the inflexions of the uterus for which the intra-uterine pessary was first applied. In his treatment of these cases he recommends that attention be paid entirely to the organic changes which may have taken place, and to the various symptoms consequent on or accompanying them; dysmenorrhœa, menorrhagia, congestion, induration, are to receive their appropriate treatment, both constitutional and local.

Ante- and retro-versions are treated in like manner, and we need not consider them at length. Intra-uterine mechanical supports are entirely rejected; he recommends the hypogastric bandage, to remove the weight of the abdominal viscera as much as possible from the pelvic organs, and limits active treatment to the removal of the accompanying congestion and other conditions upon which most of the distressing symptoms depend. This coincides with the course pursued by West, as stated in his "Lectures on Diseases of Women," in the following brief sentence: "To the best of my power, I take care of the general symptoms, and leave the misplacement to take care of itself."

The author attributes to relaxation of the vagina and of the ligaments the chief importance in producing prolapsus and procidentia of the uterus, and looks upon the congestion and ulceration usually present in these cases as a consequence of the displacement. After reduction of the inflammation, he proceeds immediately to the use of mechanical support. His remarks on the kind to be chosen are highly judicious:

"A tolerably extended experience in this department has convinced us that none of these instruments, and there never will be any, are equally adapted for all the different cases. An important support to this view is found in the varying sensibility of the parts in different females; for while some can bear without difficulty an instrument of the hardest material, which presses on and extends the walls of the vagina, another cannot endure the application of a soft sponge dipped in oil. Another proof is to be found in the fact that, in the construction of all these instruments, only one mode of origin of the difficulty has been taken into consideration, and it is therefore unadapted to cases having a different origin. We have, therefore, to keep these two points in mind in choosing an instrument to support the uterus; and, also, never to apply any apparatus which prevents the application of those remedies which may be necessary to effect a permanent cure of the disease."

In the mildest cases he recommends a sponge properly shaped, and applied when the patient is in the recumbent position; this may after a time be saturated with various astringents. The application of the latter in this manner and by injection, with those constitutional measures which may be requisite in each case, are to be adapted to the varying conditions of the case. The instrument for mechanical relief to which the author is most partial is the uterine supporter of Roser as modified by himself. It consists of a metal plate adapted to fit closely on the pubis, and retained in place by straps passing around the hips and between the thighs; to this a steel bow is attached by one end, the other terminating in an egg-shaped ebony pessary; the curve of the bow can be adapted to the patient, and by its attachment to the plate it can be lengthened or shortened, while a joint allows of some lateral motion. In some cases, where there is great relaxation of the posterior wall of the vagina, a pessary is recommended which shall widely dilate the canal in a lateral direction, and various instruments, invented by German physicians, are delineated. The gutta percha bag and tube, which is to be filled with air after its application, has been sometimes found preferable to all other means.

His experience has not been favorable to operations for the radical cure of these displacements. That consisting in removal of portions of the mucous membrane, to produce a narrowing of the vaginal canal, he has performed thirteen times, and that to afford support to the organ by causing adhesion of the posterior portions of the labia (episiorrhaphy), five times, but he has never been satisfied with the results.

"The contraction produced in the vagina yielded in a few weeks to the continued pressure of the uterus, while in the cases of union of the labia sometimes a sufficient breadth was not obtained; sometimes the united parts, in consequence of the continued pressure of the uterus upon them, gradually extended, and finally increased the opening to such an extent as to allow the protrusion of the uterus as before."

He speaks unfavorably of Pauli's method—one not likely to come into use here—and of Desgrange's mode of narrowing the vagina by the application of numerous pincettes.

Full chapters are given upon the subjects of acute and chronic inflammation of the parenchyma of the uterus. The former occurs too seldom to demand our notice. In the treatment of the latter, the chief means depended upon is the repeated application of leeches to the cervix; injections and warm baths, both containing iodine or bromine, follow sufficient depletion; and if these means fail, the use of the waters of Marienbad, Karlsbad, or Ems, is recommended. When the system shows evidence of insufficient blood-formation, iron is to be given, especially the iodide. With these means he has generally been successful in reducing the inflammation and removing the induration. He has seen nothing but injury result from a mercurial course in these cases.

We regret that the author has not entered into the questions of the importance and frequency of occurrence of ulceration of the os uteri, which has attracted so much attention within a few years past. We miss the title of Bennett's work in the bibliography preceding the chapter upon this subject, which was scarcely to be expected, since "West on the Os Uteri" is there, and Bennett's work is mentioned in other parts of the volume. However, the author must be classed with those who do *not* believe that ulceration of the os uteri is a primary affection, entailing innumerable constitutional diseases as a consequence of it; with those who do *not* believe it to be the *fons et origo* of all female diseases, although he admits that the frequency with which it occurs renders it worthy the especial study of every practitioner.

He recognizes seven forms of the disease:

1. The simplest form consists in *erosions* or *excoriations* of the mucous membrane, which so usually accompany and are caused by acute or chronic uterine catarrh. In this class he also considers the *aphthous* form of the disease, which follows the appearance of minute vesicles upon the part.

"It is this form of the disease which has been described by some English and French writers as *herpetic*, and as the local manifestations of a constitutional condition. Although we have never been able to convince ourselves that the eruption was herpetic, yet we are compelled to assent to the opinion expressed above, by some cases which have fallen under our observation, in which anomalies of blood-formation and mixture were at the bottom. For as an aphthous condition of the mucous membrane of the mouth, frequently follows derangements of digestion, exposure to damp and cold air, etc., so does the disease under consideration frequently appear after the influence of like causes; we are strengthened in this opinion by observation of a woman, otherwise healthy, who suffered a long time with aphthæ of the buccal mucous membrane, and who also had a fresh eruption upon the cervical portion of the uterus with every return of the affection of the mouth."

In and of itself these forms are of little consequence, but as the commencement of more extended and more serious forms they demand treatment. That depending upon catarrhal inflammation can only be cured by curing the disease upon which it depends; in the independent form, if there is such a form, a period of four or six weeks is necessary for the restoration of health. Local depletion by means of leeches is the first step to produce this result, then solution of nitrate of silver in the proportion of a scruple of the latter to an ounce of water, applied every five or six days. Constitutional treatment in this, and all other forms of the disease, is not to be neglected.

"Finally, it must be mentioned that in the treatment of erosions and all other ulcerations of the cervical portion of the uterus, a proper hygienic regulation of the patient is of great importance—especially the free enjoyment of pure air, the use of digestible food, and the removal of and abstinence from everything favoring hyperæmia of the pelvic viscera. The retention of the horizontal position for weeks together, as highly recommended by some, we do not consider judicious from the derangements of digestion, constipation, and imperfect blood-formation caused by it."

2. If the above simple forms of ulceration are neglected, or if they are aggravated by uncleanliness, venereal excesses, or frequent child-bearing, they take on a more severe form, called *granular*, from the peculiar appearance it presents. This never heals spontaneously, and is never a simple form, but always accompanied with considerable textural change, such as chronic induration, obstinate catarrh, of which it is sometimes the cause, though sometimes the consequence. Treatment is commenced with leeches applied to the part, and a course of aperient mineral water, continued until the accompanying hyperæmia of the organ is removed. After this, if the constitution of the patient has suffered from long duration of the disease, a tonic course of treatment is recommended, especially the preparations of iron. It is generally advisable to unite a tonic general treatment with the local, even though the latter may be depletory in its nature—a proceeding in accordance with sound general principles. Injections are of service as a means of cleansing the parts, which is indispensable to satisfactory treatment, and as a vehicle for astringents, of which solutions of alum and of muriate of iron are preferred; but caution is given not to inject the fluid with too much force, a proceeding likely to increase the congestion which we are seeking to remove, as heretofore explained.

"Almost indispensable for a favorable result of the treatment of such ulcerations is the repeated application of caustics. As to the choice of the article,—

for cases with little loss of substance and of small extent, with few granulations, and not much congestion, the nitrate of silver in substance is sufficient. If the ulcer is more extensive, with numerous granulations which readily bleed on touching, or if four or five applications of the arg. nit. have produced no good result, we must proceed to more powerful articles, among which the *liquor hydrargyri nitrici oxydati*, and the so-called Plenck's Solution (Hydrarg. bichlor. ʒii, Camph. ʒi, Alcohol ℥ii), deserve the first mention. These liquids are to be applied to the ulcerated surface with a brush or sponge, yet great caution must be taken that they come not in contact with the walls of the vagina, upon which they would produce severe effects. Also care must be taken not to apply the caustic too often, as it often happens, and we have sometimes observed it, that profuse and obstinate salivation follows neglect of this precaution."

If these means prove unavailing, the Vienna paste, and the actual cautery are to be applied. His experience does not allow him to recommend the application of tr. iodine, pyroligneous acid, or concentrated sulph. acid as caustics, nor has he seen any benefit from covering the ulcerated surface with collodion.

3. *Fungous ulceration.*—An exaggerated condition of the preceding, in which the ulcer is covered with growths, sometimes described as "cock's-comb granulations." Treatment is to be conducted upon the same general principles as above given; scarifications may take the place of leeches from the readiness with which the growths bleed, and excision is to precede the application of caustics.

4. *Varicose ulcers.*—These occur but rarely. The name is sufficiently explanatory of their nature, and the following quotation indicates the conditions giving rise to them and their treatment.

"So far we have never seen varicose ulceration of the lips of the os uteri except where, either from the presence of large tumors in the abdomen, or from disease of the heart or lungs, there was persistent obstruction to the return of blood from the pelvic vessels; sometimes we have seen it accompanied with a high degree of varicose distension of the hæmorrhoidal veins, in which cases a tolerably copious flow of blood from these vessels, almost constantly, produced a decrease of the hyperæmia of the uterus and the bleaching of the ulcer, with sometimes the total disappearance of the enlarged vessels which had been seen upon its surface."

5. *Phagedenic ulceration* is a form of disease known to the anthor only by the description of English writers, as Clark and Lever, who describe it as "corroding ulcer of the uterus." He also says that it has never been seen by any continental observer, and agrees with Kiwisch in supposing that those who have thus described it have mistaken it for ulcerated cancer with great loss of substance. In support of this view, he adduces a case which fell under his own observation and ended fatally. From the loss of substance and the peculiar appearance of the ulceration, "the pathological anatomists felt themselves compelled to reject the diagnosis of cancer, made at the clinic, and to declare the affection a phagedenic ulceration," yet a further and more minute examination proved the presence of cancerous infiltration in the diseased mass and in the neighboring lymphatic glands. He also quotes Rokitansky, who describes the similarity between destroying ulceration of the cervix uteri, and phagedenic (cancerous) affections of the integument.

6. Upon the subject of *syphilitic ulceration* of the cervix uteri, he disagrees with Kiwisch, who points out two forms of this kind of ulcer. Neither from reading nor observation has he been enabled to distinguish the difference between syphilitic and simple ulceration of the uterus; he quotes German writers who have enjoyed the widest fields of observation, to show how rarely primary syphilitic sores are found on the cervix, while erosions and granulated ulcerations are constant accompaniments of vaginal and uterine catarrh, diseases so frequent in those who pursue a life likely to make them the subjects of chancre.

7. *Cancerous and tubercular ulceration* are considered in connection with the diseases with which they are only found, and they make up the last of the seven divisions under which the author considers these affections. Our only excuse for occupying so much space with them, must be the desire to present his views in full, upon a subject at present receiving so much attention as the one in question.

The subject of leucorrhœa is considered under the heads of acute and chronic catarrh of the uterus. As an independent disease its existence is ignored, and in this the author agrees with Rigby, who remarks in his lately published work, "Constitutional Treatment of Female Diseases," p. 80, "To consider leucorrhœa, *per se*, as a peculiar disorder or disease, requiring specific treatment, appears to be as unpractical and erroneous as it would be to treat of an affection called *expectoration*." Yet the author does not, of course, maintain that this catarrhal inflammation is always the primary disease, but points out its frequent dependence upon textural changes, such as polypi, tumors, and chronic induration of the uterus. The reaction of the discharge is depended upon to distinguish the uterine from the vaginal forms of the disease—being alkaline in the former, and acid in the latter, as pointed out first by Whitehead, and since substantiated by Tyler Smith. In the treatment of this disease he does not recommend or countenance any plan which is limited to either local or constitutional means; the two must be united to produce a cure. Where the inflammation of the mucous membrane, which causes the discharge, is but the result of some form of organic disease, of course treatment must be directed to that; where it is simple and primary, the extent to which local depletion and aperients are to be carried, will vary with the more or less acute character of the case. Aperients are preferred in the form that nature presents them in various mineral waters; and such a course of life as is generally led at watering-places is highly recommended. Injections should be commenced lukewarm and gradually decreased in temperature, and astringents added. In severe chronic cases cauterization of the interior of the cervix and body of the uterus with the solid nitrate of silver is recommended, which is to be repeated every twelve or fourteen days for six or eight weeks. If these means fail, he has no faith in the use of the preparations of iodine, catechu, copaiva, rhatany, kino, or like remedies.

Anomalies of menstruation are considered under six different heads. 1, too early appearance of menstruation; 2, delayed appearance; 3, too early, and 4, too late cessation of the flow; 5, want of the most important symptom of the menstrual hemorrhage, during the period of sexual life; 6, menorrhagia; 7, dysmenorrhœa. These chapters are preceded by one upon the subject in general, its influence upon the general health, etc. From the pains he takes to impress upon the reader the idea that suppression of menstruation is more frequently a consequence than a cause of deranged general health, and that a retention of the usually excreted fluid, cannot have any injurious effect upon the system at large, we draw the inference that the physiology of the function is not as well understood in the land of Bischoff as it deserves to be.

We pass over the chapters on uterine tumors, and the second and third divisions of the work, the one upon the pathology and diseases of the ligaments of the uterus, the other upon those of the Fallopian tubes, and pass on to the fourth, on ovarian diseases, as far more worthy of the space which remains at our disposal. This is a subject to which, probably, more attention is at present directed than to any other in this branch of medical science. Peculiar difficulties surround the investigation of diseases of the ovaries, on account of their situation, and the extent to which morbid processes may advance in them before they attract attention; while a decision upon the best of several modes of treatment is embarrassed by the conflicting nature of the testimony given by the few who have enjoyed advantages of observation sufficient to render their opinions of value.

What is the extent and character of the influence which disease of the ovaries exerts upon menstruation? This is a question very variously answered by different authors. Some state that the menstrual flow under these circumstances is always suppressed or deranged; others that this is only generally the case; others that it *may* occur; while one well-known writer sees the cause of all anomalies of the function, without exception, in ovarian disease. Our author allows a certain value to variations from normal menstruation in forming a diagnosis of disease of the ovaries, but the following quotation will show his opinion of the "ovarian theory:"—

"It is generally understood that the state of the menstrual hemorrhage, singly and alone, allows of a judgment upon the regular or abnormal progress of ovulation. But that this cannot be exact and reliable, is evident from the fact that the maturation and expulsion of the ovum can proceed regularly; and nevertheless many anomalies of the external sign, the menstrual flow, may occur. On the contrary, it is well known that the ovaries sometimes exhibit very considerable organic changes, and that nevertheless menstruation has not exhibited any disturbance; this is even observed in diseases of both ovaries, so long as yet a part remains which contains a single Graafian vesicle in which the ovum can proceed to its development."

The bibliography preceding the chapter on the important subject of ovarian tumors is fuller of English names than that belonging to any other subject in the book. It contains, we believe, the name of every writer upon this subject of note in Great Britain. The work of Clay, published last year ("Complete Handbook of Obstetric Surgery"), is not, however, included,—a fact which is to be regretted, since his experience was far greater than at the time of publication of the work mentioned by Scanzoni (1842), and his statistics bear strongly upon the point of extirpation.

Ovarian tumors are divided by the author into two classes, cystic and solid. The first is subdivided into simple cysts, united cysts, colloid tumors, cysto-sarcoma, and cysto-carcinoma; the second class into fibrous, enchondromatous, cancerous, and melanotic. By simple cystic disease he means the true dropsy produced by dilatation of a Graafian vesicle, while the united cysts arise from excessive growth of the fibrous tissue of the stroma of the ovary, the resulting cavities afterwards becoming filled with fluid of different consistence. It is this form of disease that sometimes presents instances of teeth, hair, bones, &c., in the tumor.

Colloid disease must not be confounded with the cancerous disease of the same name; the author in this follows Virchow, who applies the term to a tumor made up of a multitude of cysts filled with a tenacious, gelatinous fluid, and well known under the name of *multilocular cystic dropsy*, but differing widely in its origin, progress, and termination, from dropsy. The views of Virchow upon the pathology of this form of disease may be found in Rigby's late work on the "Constitutional Treatment of Female Diseases."

The relative frequency of occurrence of the different forms of tumor given above is represented in a table of forty-one cases, in which a post-mortem examination allowed of a certain decision upon the subject; these were the fatal cases which occurred in ninety-seven of ovarian tumor, found in an accurate record of 1823 cases of female disease examined by the author. Of the 41 cases, there were, of simple cystic dropsy, 13; united cysts, 12; colloid tumor, 9; cysto-sarcoma, 5; and cysto-carcinoma, 2. The following table shows the side upon which the disease occurred, compared with the results given by Lee and Chereau:

| | No. of Cases. | Right Side. | Left Side. | Both Sides. |
|---|---|---|---|---|
| Lee, . . | 93 | 50 | 35 | 8 |
| Chereau, . | 215 | 109 | 78 | 28 |
| Scanzoni, . | 41 | 14 | 13 | 14 |

The ages at which the patient first observed symptoms of the disease in ninety-seven cases are stated in another table; suffice it to say, that of these, sixty-seven were between the ages of thirty and forty years.

Of the ninety-seven patients, fifty-one had never conceived; and of forty unmarried, there were sixteen with the genitals in a virgin condition; from which the author concludes that entire abstinence from sexual enjoyment must be considered as in some measure a cause of the disease. But he finds it difficult to decide whether sterility is more frequently a cause or a consequence of the disease.

Upon the subject of diagnosis of these tumors we extract the following paragraphs on auscultation:

"If the large vessels at the entrance of the pelvis are compressed by the tumor resting upon them, or if there are developed within the tumor itself large vessels, especially arteries, it may be that on auscultating the lower part of the abdomen, particularly the inguinal region, that vascular murmurs are heard, which are identical with those murmurs known as uterine, which occur within the pregnant uterus. Notwithstanding the occurrence of these murmurs in cases of ovarian tumor is denied by some, for instance Kiwisch, yet we can assure our readers that we have perceived them with perfect certainty in several, although not in numerous cases, where the post-mortem examination afterwards proved the existence of ovarian tumor, and our observation has been supported by that of other listeners; we will mention, however, that we could never discover these sounds in cases of simple cysts, but that it only occurred in connection with more compound tumors provided with a more developed vascular system.

"As a second auscultatory symptom, perceptible in the lower part of the abdomen, must be mentioned the friction-sound, which is to be heard at some points of the abdominal walls during the movements of respiration, and those resulting from change of position of the patient. It arises undoubtedly from the rubbing together of the rough outer surface of the tumor and inner surface of the abdomen. This roughness is the result of exudations which have been poured out on these surfaces, and which were found present in every fatal case we examined. Near these roughened surfaces there were found also, without an exception, more or less numerous and intimate adhesions of the tumor to the anterior wall of the abdomen, so that we believe we do not go too far when we maintain that this friction is to be considered as an almost reliable sign of the presence of adhesions. We will also mention that the roughness of the surfaces sometimes reaches such a degree that the hand laid upon the surface easily perceives the friction."

We are told in a late work on female diseases that ascites and fibrous tumors are the only diseases likely to be confounded with ovarian tumors, although pregnancy is afterwards alluded to as being possibly mistaken for them. Compared with such a statement, the fulness and minuteness of our author upon the differential diagnosis of these tumors deserves notice. Besides the conditions just mentioned as likely to render a diagnosis difficult, he enumerates organized exudations in the folds of the peritoneum, retroverted uterus, extra-uterine pregnancy, subperitoneal cysts, accumulation of fæces in the cœcum or sigmoid flexure of the colon, flatus, exostosis and fibrous tumors of the pelvis, enlargements of the spleen, kidneys, and liver, and distended bladder.

Enlargements of the kidneys and liver the author says are very unlikely to be mistaken for ovarian tumor, yet an instance has occurred in this country where an ovarian tumor was called an enlarged liver even after removal from the body. It would seem that a distended bladder could not easily be mistaken for ovarian dropsy, and yet many authors mention the fact that this organ has been pierced by the trocar introduced under such a misapprehension.

We proceed to notice the different modes of treating the disease for a radical cure. The author has no faith in general treatment, although he has seen some tumors remain stationary and a few decrease in size during a course of alteratives, and the use of some of the mineral waters. Simple puncture of the cyst, with evacuation of its contents, is an operation he is opposed to, as not being entirely free from danger, and as very unlikely to fulfil the desired object, although he has seen two cases where a permanent cure resulted from it. The attainment of a cure by this means is only possible where the cyst is single, and improbable then from the impossibility of tapping it low enough to drain it entirely of fluid, a condition almost essential to obliteration by adhesion of its walls. He counsels this mode of treatment only in cases where, from the position of the tumor, it is impossible to put in force the next following mode of puncture, and considers those cases where the tumor does not exceed the size of a man's head as most likely to result in a cure. As there is a laudable disinclination to proceed to operative interference until compelled to, we think the tumor will generally be allowed to surpass the size, and when this is the case, the author is not in favor of tapping until the patient is highly incommoded by it, or until danger is occasioned by its pressure on vital organs.

"If the aforesaid be kept in mind, and the large ovarian tumors considered as true '*noli me tangere*,' the number of cases in which paracentesis abdominis is performed for them will be far less than has been heretofore the case; and we cherish the conviction that with the increasing rarity of this operation the lives of these unfortunate patients will be longer spared, than in the time when the presence of hydrops ovarii was alone the indication for the use of the trocar. We refer those of our readers who are interested to the tables of Lee, already quoted, which show that paracentesis, as a palliative in ovarian dropsy, is a very dangerous proceeding; that when recourse to it has once been had it must be frequently repeated; that the relief from one operation to another constantly decreases, and the danger as constantly increases."

The dangers of the operation are then enumerated at length, the chief of which are inflammation and suppuration of the sac, peritonitis, hemorrhage from wound of a vessel ramifying on the cyst, on the walls of the abdomen, or of the omentum, which last occurred under the author's observation, and terminated fatally. Sometimes the operation is followed by nervous depression, convulsions, and collapse, from sudden evacuation of the fluid, and consequent tension of organs united by adhesion.

There is nothing peculiar about his mode of operating, except that he allows the puncture to be made through the umbilicus under certain circumstances,—a proceeding which is cautioned against by most other writers; and he mentions a patient in the hospital at Wurzburg who had tapped herself, by opening an ovarian cyst, which protruded at this point, with a razor, no less than twenty-two times in three years,—a case far more wonderful to relate than creditable to the regulations of the hospital.

Puncture of the cyst through the walls of the vagina is the favorite treatment of the author,—one which he prefers to all others, and would always put in force were it always practicable; but, of course, it can be performed only in cases where the situation of the tumor is so low as to bring it in contact with the upper part of the vagina. A trocar is to be introduced into the cyst, and if the fluid is too viscid, the puncture may be carefully enlarged to an incision an inch or inch and a half in length, and tepid water may be injected to favor its flow; after evacuation, the canula is to remain eight or ten days. The advantages of this mode of operation in procuring a more perfect removal of the fluid are obvious, but we cannot see upon what grounds the author claims that it is less likely to be followed by inflammation of the sac and peritoneum. Such inflammation arose in three out of fourteen cases operated upon by him; but a radical cure followed in all three, as was proved by many years' subsequent observation. Of these fourteen cases, he is certain of a perfect cure in eight; in two the cyst filled again within a few weeks; three withdrew from observation, and one died of typhus two months after the operation, but no post-mortem examination was held. He has had no fatal case, but seen one in Kiwisch's practice; he has had one case of cure following the second puncture by this method.

Puncture of the cyst, followed by injections of irritating fluids, is at present attracting considerable attention, and the plan is now being tested by some of the best men of Great Britain, whose decisions we look for with the greatest interest. It is a plan which finds no favor with the author. His criticisms are directed against Boinet's plan, which consists in puncture and evacuation, the repeated injection of solutions of iodine, and iod. potassium, and the retention of a catheter in the cyst, which, after the seventh or eighth day, is to be frequently changed. The objections are, that by the gradual contraction of the cyst the catheter may slip out of it, and the next injection be made into the abdominal cavity; that adhesion does not invariably take place between the cyst and abdominal walls, and that the same accident may occur on changing the catheter; that we cannot always be sure of a simple cyst; and that it is impossible to estimate the amount of inflammation which will follow. Death sometimes follows simple puncture, and is far more likely to result from injection of such fluids, and he gives a case in his own practice, where he put it in force for a simple cyst which had repeatedly filled, and in which death followed on the fifth day after the operation.

Incision of the sac, as recommended by Ledran, Velpeau, and others, he is also opposed to, as in cases of simple cysts puncture is far preferable, from the smaller injury inflicted on the peritoneum. The latter also affords sufficient opportunity for escape of the contents, if fluid, while, if they are viscid, the disease belongs to the multilocular class, and evacuation cannot be procured. He fears the dangers of peritonitis from acupuncture, application of caustics, &c., which have been recommended preparatory to this operation.

Excision of a portion of the sac is considered more hazardous, and less likely to yield a favorable result than incision. This mode of treatment is preferred by Isaac Baker Brown in his work on "Some Diseases of Women admitting of Surgical Treatment," yet it did not originate with him, as is implied by our author, having been proposed, at least, by Blundell. We quote his remarks, which are expressed with not a little tartness, and with no excess of respect for his English professional brethren.

"Although we will not insist that the operation in itself is a very dangerous one, and upon this point the opposed statements of the well-known little-reliable English physicians cannot lead us astray, even if we place the danger entirely out of view, yet we would call to mind the fact that the walls of large cysts are often very vascular, and that a wound of these vessels would necessarily cause dangerous hemorrhage into the cavity of the abdomen or of the cyst. If Brown maintains that his mode of operating is only to be followed where the walls of the cyst are not vascular, he has not answered the question how the greater or less vascularity of the cyst is to be ascertained before opening the abdomen. It is also to be considered that excision of a portion of the wall of the cyst will cause violent inflammation of the same; and, if this is the case, it cannot be guaranteed that the resulting exudation will not be ichorous or purulent, thereby inevitably causing violent peritonitis; in short, so long as favorable results of this operation are not reported by more reliable authority, so long we shall take care not to follow Baker Brown's proposed plan."

To those acquainted with Mr. Brown's work it will be evident that Scanzoni has not done him justice, since Mr. Brown recommends an incision through the abdomen long enough to enable the operator to distinguish the number and position of the vessels of the sac, and directs the part to be removed in such a manner as not to interfere with them.

The remaining operation for the cure of ovarian disease to be noticed here, and the most important, is extirpation—one which has most ardent friends and bitter enemies. There is a natural disinclination to attempt an operation of such magnitude and danger, unless to rescue a patient from immediate peril, and her danger from the operation is increased by delaying it until it is a matter of necessity. On the other hand, the operation has been successful, and in a respectable proportion of cases; indeed, a late writer claims that few, if any, of the great operations of surgery can show so large a percentage of successful results; "nor, when successful, can they boast of that great and peculiar feature which belongs to ovariotomy, viz., that, with few exceptions, whenever the patient recovers, her recovery is complete and permanent."* It is not our province here to give the arguments for and against ovariotomy. With our present knowledge, it is impossible to decide the superiority of any of the different modes of treatment, and until we shall have collected the experience necessary to decide the questions at issue upon extirpation, let us not degenerate into partisans, however strong the arguments upon either side may appear.

Before giving the views of the author upon the operation, we quote the following sketch of its history. After stating that Schlencher was the first who proposed it—at what date, however, is not mentioned—he says it was considered by Tozzetti, 1752; Willius, 1734; Peyer, 1751; and by De Haen (*de ratio medendi*), who judged that it was dangerous even to think the operation possible! and by Van Swieten, who was of opinion that a favorable result might be obtained in some cases.

"So far as we know, C. Aumonier, of Rouen, in 1781, was the first who performed this operation; it was upon a patient twenty-two years of age, and was successful. He was followed by Eph. McDowell, in America, who, in the year

* Rigby, Constitutional Treatment of Female Diseases, p. 249.

1809, also operated most successfully, and afterwards repeated it several times. From this time on, extirpation of the diseased ovary gained friends, and it was principally English and American surgeons who recommended it, and sometimes successfully performed it. The French surgeons were less favorable to it, and even in 1847 Velpeau (Gaz. des Hop. No. 99) pronounced these operations to be the result of foolhardiness; that they should be opposed with all our influence, because they were the offspring of folly, and that it was to the honor of French surgery that such proceedings were not countenanced by it. In Germany it was principally Kiwisch who forwarded the operation by word and deed, and it was principally his authority which influenced other surgeons of his country to follow his example, so that within the last two decennial periods the Fatherland has seen numerous cures as the result of this operation."

Rigby gives the honor of the first operation to Jefferson, of Sussex, performed in 1836! yet Lizars reported a case as early as 1825!

We append our author's views upon the operation, which need no introduction and are not likely to be misunderstood:

"We hold the extirpation of ovarian tumors as a surgical adventure, which, when it succeeds, must be thankfully glorified by the patient and gaped at by the public. It is an adventure, because it is never possible for the surgeon, according to experience, to indicate the result of the operation with that amount of probability which he can that of other capital surgical operations; for experience has shown that operations of this kind, undertaken under the most favorable circumstances, performed by the most skilful hand, and without the complication of accidents, have resulted in the death of the patient a few days or hours after its completion, or even during its performance. Even if the published statistics of the operation do not speak for its absolute fatality, or even if they prove a not inconsiderable number of cures, yet they can in no wise induce us to favor a proceeding which was rapidly followed by death, in 5 cases out of 6, operated on by a Langenbeck, and in 4 cases out of 5, by a Kiwisch; for if we allow that of these 11 patients 2 were truly healed, yet no one can deny that the lives of the remaining 9 were considerably shortened; and if men so renowned as these have to lament such an extremely unfavorable result of their operations, will not the courage of the less experienced sink? But if it is replied, that the results of ovariotomy have heretofore been so unfavorable because the operation is generally only undertaken when the tumor has attained a considerable size and adhesions have taken place between it and neighboring parts, when the powers of the patient have given way, and the general organism has been involved; if this be replied, then, we would ask, what considerate, honest-minded surgeon would undertake an operation so extremely dangerous to life at a time when the tumor is yet so small as to occasion the patient inconsiderable, if any, inconvenience or suffering; at a time when it is impossible for the surgeon to decide whether the disease will not remain stationary, or only make such gradual progress as to spare the life of the patient many long years? Then we are compelled to operate only under the above-mentioned unfavorable circumstances, and thus have to expect in all probability an unfavorable result, unless the pressing entreaties of the patient induce us to proceed to the attempt at an earlier period of the disease. And some writers have, therefore, expressed the opinion that a surgeon, even if he is not inclined to favor ovariotomy, is justified in performing it when the patient overwhelms him with entreaties, and, in spite of all representations, remains fixed in the resolution to die rather than bear the sufferings of her disease any longer. We found ourselves once in such a situation, and gave way to the desires of the patient; but we are firmly resolved never to do it in the future; for if it is not to be denied that every person has the right, to a certain extent, to decide upon his own body, it is also certain that to this right bounds are set, and it must be called foolhardiness for a patient, in order to escape the pains of her disease, to allow an operation upon herself, which with the greatest probability will end in death. But in this the patient may be partly excused, for her judgment is influenced by the pains endured, but the surgeon is not to be excused who lends himself as the instrument even of an involuntary self-murderer.

"From the foregoing, it will be seen that we cannot speak in favor of ovariotomy, and we renounce the glory of having successfully performed such an operation, until our above-expressed opinion upon its almost absolute fatality has received a convincing statistical refutation."

The fifth division of the work treats of the Pathology and Therapeutics of Diseases of the Vagina. We confess we turned to the chapter upon vaginal fistulæ with a great deal of interest, and suffered not a little disappointment upon reading it. The bibliography not only contains no American work, but also none in the English language,—a translation of Chas. Bell's Principles of Surgery is the only Anglo-Saxon authority mentioned! We do not think we had any right to expect that the brilliant operation of Dr. Bozeman, published in this country in May, should be known in Germany at the time the author's work went to press, in the fall of the same year, but there is just cause of complaint in the omission of the name of Dr. Sims, whose operation was one of the most decided steps in advance ever made in this branch of the healing art, and which has now been so long before the profession as to leave no excuse for a writer occupying the station that Scanzoni does. Herein we think we find just cause for congratulation on the extent to which the profession in this country is informed upon every new mode of treatment or new operation which may arise in any part of the world, and we do not think a similar instance to that mentioned above can be found in our whole country, however little the labors of the profession here may be esteemed in transatlantic countries.

The operation preferred by the author for vesico-vaginal fistula is Jobert's, with some improvements introduced by Simon. He records two cases which he has cured by the application of caustics; but we judge that his experience with operations of this kind has not been extensive. This division contains a long and interesting chapter upon catarrhal inflammation of the vagina, in which is found everything relating to leucorrhœa not included in the division upon diseases of the uterus.

The Pathology and Diseases of the External Organs of Generation forms the sixth division of the work. The subject of rupture of the perineum need alone detain us here, although all the malformations, and retarded and excessive development of each separate portion, are duly treated of. Believing in the good old maxim about the relative value of prevention and cure, the author recommends a mode of proceeding to avert this accident in which, we dare affirm, he stands alone in the world, and presents a striking instance of the extent to which men of high standing in the profession may depart, upon some point, from the plainest dictations of pathology, to say nothing of sound sense:

"While we refer to treatises upon obstetrics for the general prevention of rupture of the perineum, we will here only briefly mention, that we know of no surer mode of avoiding this accident than the artificial enlargement of the vulva, which is to be effected by two incisions, from four to five lines deep, made in its posterior circumference, at the moment when the presenting part of the child is about to be expelled through it,—a proceeding which we have put in force more than a hundred times [mehr als hundertfältig erprobt], and which is fully described in our work on obstetrics."

Undoubtedly, a most *sure* mode of prevention! but if the author has committed this piece of interference more than a hundred times, how many times has it been unnecessarily performed! It is with the deepest regret we record so great a blemish in a work otherwise of great practical value.

For the treatment of recent cases of rupture of the perineum the author recommends the immediate application of sutures, perfect cleanliness, and the lateral position of the patient; for old-standing cases he lauds highly the plan of Mr. Brown.

The space we have already occupied precludes any notice of the seventh and last division of the work, on the Diseases and Pathology of the Mammary Gland; any remarks upon these subjects are the less necessary here, as they have been fully treated in a review of Velpeau's work, contained in a late number of this journal. We need only say that this part of the book compares favorably with what precedes it.

We bring our notice of Scanzoni's work to a close. In presenting it to our readers, we have indulged more in extracts than we should have done had it been in our own language and accessible to all, being desirous of affording an opportunity to judge of its merits, although we believe the fame of the author more deservedly rests on his work on Obstetrics. Germany has a rich treasure of medical literature; the science is there cultivated with ardor; the profession there contains some of the most illustrious names in the annals of science; and we congratulate ourselves upon the opportunity of bringing before the notice of our readers one of the works of a writer who occupies a high position in a land where sound education and long experience are the necessary qualifications of an author.

